# Online self-assessment of cardiovascular risk using the Joint British Societies (JBS3)-derived heart age tool: a descriptive study

**DOI:** 10.1136/bmjopen-2016-011511

**Published:** 2016-09-01

**Authors:** Riyaz S Patel, Catherine Lagord, Jamie Waterall, Martin Moth, Mike Knapton, John E Deanfield

**Affiliations:** 1 National Centre for Cardiovascular Prevention and Outcomes, Institute of Cardiovascular Sciences, University College London, London, UK; 2 Bart's Heart Centre, St Bartholomew's Hospital, London, UK; 3 Public Health England, London, UK; 4 NHS Choices, London, UK; 5 British Heart Foundation, London, UK

**Keywords:** Digital health, Cardiovascular Risk, Risk factors, Cardiovascular Prevention, Self Assessment

## Abstract

**Objective:**

A modified version of the Joint British Societies (JBS3) ‘heart age’ tool was introduced online to broaden access to personalised risk assessment to the general population and encourage participation in the National Health Service (NHS) Health Check programme. This study reports on its early uptake and the profiles of those who used the self-assessment tool to determine their own cardiovascular risk.

**Design:**

Observational, retrospective analysis of online tool use.

**Setting:**

Between February and July 2015, user data collected from the NHS Choices website, where the tool was hosted, were analysed anonymously using standard analytic packages.

**Results:**

The online tool landing page was viewed 1.4 million times in the first 5 months, with increased activity following limited media coverage. Of the 575 782 users completing the data journey with a valid ‘heart age’ result, their demographic and risk factor profiles broadly resembled the population of England, although both younger users and males (60%) were over-represented. Almost 50% and 79% did not know or enter their blood pressure or total cholesterol values, respectively. Estimated heart age was higher than chronological age for 79% of all users, and also for 69% of younger users under 40 years who are at low 10-year risk and not invited for NHS Health Checks.

**Conclusions:**

These data suggest a high level of public interest in self-assessment of cardiovascular risk when an easily understood metric is used, although a large number of users lack awareness of their own risk factors. The heart age tool was accessed by a group not easily reached by conventional approaches yet is at high cardiovascular risk and would benefit most from early and sustained risk reduction. These are both important opportunities for interventions to educate and empower the public to manage better their cardiovascular risk and promote population-level prevention.

**Strengths and limitations of this study**

- The first study on the uptake and use of the Joint British Societies (JBS3)-derived ‘heart age’ tool by members of the public in the UK for self-assessment of cardiovascular disease (CVD) risk.
- Academic and public health collaboration with full access and analysis of anonymised online data for over 500 000 users, collected through the National Health Service (NHS) Choices website.
- Unrestricted access to the tool for the public, thus enabling assessment and reporting on the characteristics of users choosing to self-assess risk.
- Through analysis of online entries, the study was able to demonstrate the levels of knowledge members of the public have about their CVD risk factors.
- As this work describes online behaviour only, further study is required to explore the impact of self-assessment of risk using the ‘heart age’ on perception of risk and behaviour change.

## Introduction

Despite a 44% reduction in cardiovascular disease (CVD) mortality in the UK following the introduction of government health policies, novel treatments and public health initiatives over the last decade, CVD still contributes to a third of all deaths each year, while rising levels of obesity, diabetes and an ageing population threaten to reverse these gains.^1–3^ In response to this challenge, and understanding that CVD begins early in life, the National Health Service (NHS) Health Check programme was introduced in 2009 for those aged 40–74 years to identify individuals at high risk for CVD at a relatively young age and encourage them to modify their lifestyle with or without therapeutic intervention to prevent onset of disease later in life.^4^ Among criticisms of the approach was the use of a 10-year risk estimate, which is not intuitive for the public to understand and which may provide false security for younger people who despite substantially elevated risk factor levels often fall below the 10% threshold for intervention which is currently recommended.^5^ ^6^

In 2014, the Joint British Societies for the prevention of CVD published guidelines (JBS3) that introduced a novel risk calculator for estimation and communication of CVD risk, based on the lifetime impact of risk factors on CVD using the QRisk Algorithm.^7^ ^8^ This included estimation of a ‘heart age’ and also permitted demonstration of the benefits of interventions, especially when they are introduced at an earlier age and are sustained. In February 2015, a public facing version of the JBS3 ‘heart age’ tool was developed in conjunction with Public Health England (PHE), the British Heart Foundation (BHF) and NHS Choices (The government run health website for the NHS) as part of the wider response to the Department of Health's CVD Outcomes strategy with its emphasis on prevention and risk factor control.^9^ This was launched on the NHS Choices website aiming to empower individuals proactively to manage their risk factors and potentially to improve NHS Health Checks programme participation.^4^ ^10^

There has been tremendous enthusiasm from the public with over 1.4 million hits to the website in 5 months. Here, we report on the characteristics of individuals who chose to assess their own CVD risk online including their demographic and risk factor profiles, together with their knowledge of their risk factors and subsequent online actions. Our findings have potential implications for strategies designed to improve public understanding of personalised CVD risk and to enhance delivery of national CVD prevention initiatives.

## Methods

Data generated through user interaction with the NHS Choices website page website hosting the heart age tool is continuously captured and stored in an analytics database. These data were made available to the authors for aggregate analysis and reporting.

### Study population

Data from all users were collected from the launch date of the tool (11 February) to 9 July 2015. Data were not restricted to any condition for estimation of website hit counts, but detailed descriptive analysis was limited to those who completed the user journey yielding a valid ‘heart age’. By using the tool, users consented to providing data items online and use of their data in an anonymised fashion. Postcode (location) data were requested to estimate the Townsend score for deprivation, a means of assessing affluence based on residential location,^11^ but was not stored. No other personal identifiable data including IP address were requested or captured.

### Data sources

*JBS3 heart age tool*: Full details of the JBS3 risk calculator have been published previously.^7^ The calculator estimates a ‘heart age’, through multivariable modelling which is referenced to someone of the same age, gender and ethnicity with optimal risk factors (eg, non-smoker, blood pressure (BP) 120 mm Hg). Although freely available online (http://www.jbs3risk.com), the JBS3 calculator was designed for use by healthcare professionals to communicate risk and treatment opportunities to their patients.

We developed a public facing version of the tool in partnership with PHE and the BHF to allow members of the public to assess their own CVD risk. Of the novel metrics, ‘heart age’ was considered the most user-friendly and acceptable for public use and interpretation. This heart age tool has six pages, screenshots and full descriptions of which are presented in table 1. Users are guided through each step, requesting basic details such as age, gender, ethnicity, postcode, before moving onto risk behaviours and then measures including height, weight, total cholesterol and BP. Questions are presented in a simple yes/no format with open fields for numerical inputs. A choice of units is available to facilitate data entry. The end result, assuming valid data entry at each step, is generation of a ‘heart age’, defined as above. Users with a history of established CVD were not eligible to use the tool. Details of the data items requested at each stage are listed in online supplementary methods.

**Table 1:** Website visitors and completion numbers at each page

Stage in journey	User starting N	User dropout N	Screenshots
Page 0 Reach heart age tool website	1 439 486	355 224	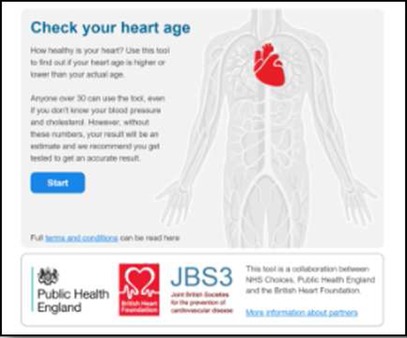
Page 1 Enter demographics, ask for previous CVD**	1 084 262	184 359	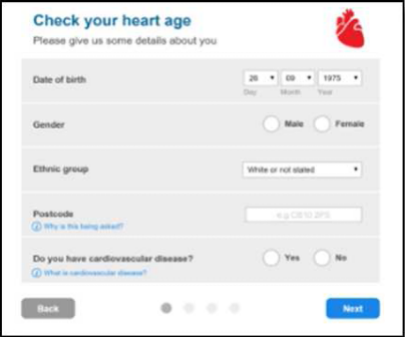
Page 2 Smoking status, height and weight	899 903	61 132	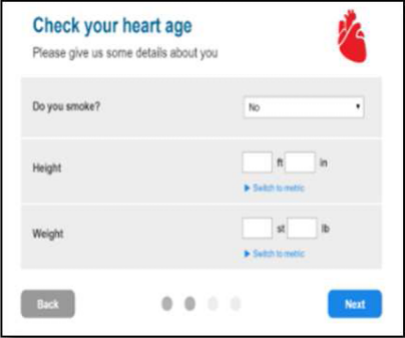
Page 3 Cholesterol and blood pressure	838 771	129 857	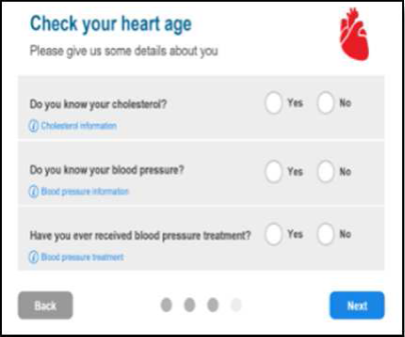
Page 4 Medical conditions	708 914	133 132	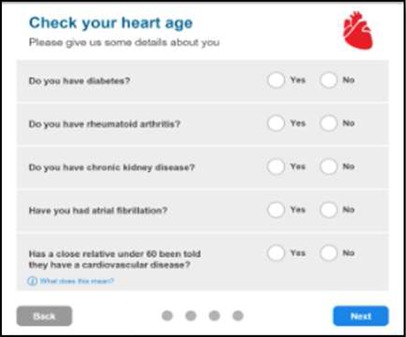
Page 5 Result	575 782	–	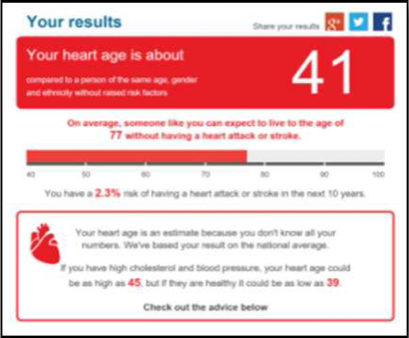

CVD, cardiovascular disease.

10.1136/bmjopen-2016-011511.supp1 supplementary data

This modified heart age tool, hosted on the NHS Choices website, was launched on 11 February 2015 to complement the NHS Health Check programme (https://www.nhs.uk/tools/pages/heartage.aspx).^4^ After estimation of a ‘heart age’, users were invited to follow web links that offered advice on reducing specific risk factors aiming to trigger behaviour change or increase uptake of the NHS Health Check programme.

### Data analysis

All variables were analysed as either continuous or categorical traits with summary statistics generated as counts, proportions (%), means (SD) or medians (IQR). Thereafter, detailed analysis was only performed for those who completed the user journey yielding a valid heart age.

*User profiles*: Age was categorised by 10-year groupings and also assessed as 40–74, reflecting the NHS Health Check age range. Postcode was provided in a subset of the data and used to generate the Townsend score of deprivation.^11^ Electoral ward-level Townsend deprivation scores were based on the 2001 Census.^12^ Knowledge gaps were estimated based on the proportion of people who did not enter numerical values for height, weight, BP and total cholesterol. Risk factor prevalence was estimated as proportion of users reporting current smoking, hypertension (treated or >140 mm Hg systolic BP (SBP)), hypercholesterolaemia (total cholesterol >5 mmol/L), diabetes and obesity (body mass index (BMI)≥30 kg/m^2^). Comparison to England population estimates was made using Census and HSE survey data.^13^

Ethical approval was not required for this anonymised data analysis. All analyses were performed using Stata V.13 (StataCorp).

## Results

Between 11 February and 9 July 2015, the JBS3 heart age tool landing page was accessed 1 439 486 times with a median of 1443 hits per day (IQR 1128–2697). Tool use fluctuated over time with spikes in activity corresponding to media coverage and press releases. Most noticeably, following national newspaper coverage, the site was accessed over 550 000 times in 1 day alone (see online supplementary figure S1).

In total, there were 592 571 completed data journeys. Among those not completing the journey (n=846 915), the majority of dropout (n=355 224) was at the front page (‘splash’ screen) with a steady attrition rate at each subsequent page (table 1).

The following descriptive data are for the 575 782 users completing the data journey and yielding a valid ‘heart age’. This excludes 16 789 users in whom a heart age could not be calculated due to out of range data, or who self-reported prior CVD (n=14 249).

### Heart age tool users and population representativeness

**Figure 1:**
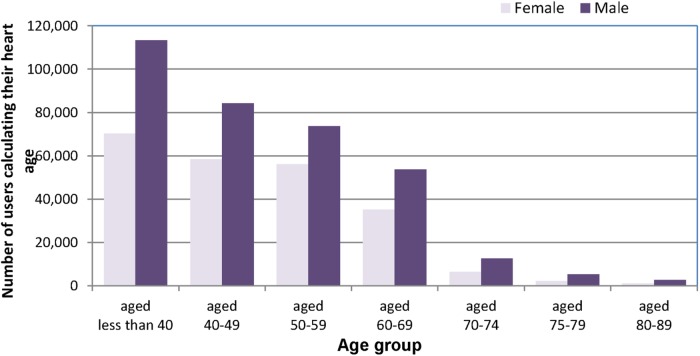
Age and gender distribution by 10-year categories for users of the Heart Age tool that completed the user journey yielding a valid heart age.

*Age*: There was an inverse trend with age and tool use, with nearly a third of users <40 years (30.6% women, 32.8% men). Two-thirds of users were between the ages of 40 and 74 (68% women and 64.9% men), the target age for the NHS Health Checks (figure 1), with only 1.4% of women and 2.3% of men 75 years or over (see online supplementary table S1). The user 5-year age profile correlates well with the England 5-year age profile from age 25 to 85 years (R=0.95; Office for National Statistics (ONS) 2014, data not shown).^14^ Overall, among tool users, there were more people aged 30–60 and fewer older people >75 years (2% of all users).

*Gender*: Initially there were more female online users (52%), although this changed appreciably after media releases which enhanced awareness of the tool and its application (see online supplementary figure S2). Overall, among those completing the data journey, 61.1% of risk estimates were for male participants.

*Ethnicity*: The majority (86.94%) of users self-reported ethnicity as white (also the default option if left blank). Of the remainder, users identified as ‘other’ (3.73%), ‘Indian’ (3.33%), ‘other Asian’ (2.87%), ‘Chinese’ (1.05%) and ‘Pakistani’ (0.85%). There were substantially fewer ‘black African’, ‘black-Caribbean’ and ‘Bangladeshi’ users (0.62%, 0.40% and 0.21%, respectively). The majority (86.9%) of users self-reported ethnicity as white (also the default option if left blank). Of the remainder, users identified as ‘other’ (3.8%), ‘Indian’ (3.3%), ‘other Asian’ (2.9%), ‘Chinese’ (1.0%) and ‘Pakistani’ (0.9%). There were substantially fewer ‘black African’, ‘black-Caribbean’ and ‘Bangladeshi’ users (0.6%, 0.4% and 0.2%, respectively).

**Figure 2:**
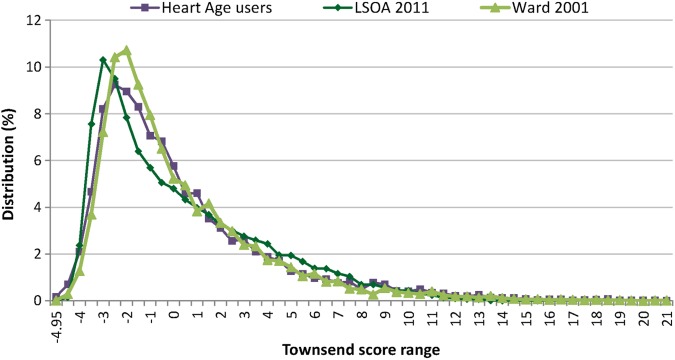
Townsend profile of heart age tool users showing a similar distribution to that from Census data for England, suggesting good representativeness of the sample covering the full deprivation range. Comparison with lower LSOA based on Census 2011 and with deprivation profile of electoral wards (based on Census 2001). LSOA, layer super output areas.

*Postcode*: Only 39.4% (n=226 824) entered a valid England postcode with which a Townsend score for deprivation could be estimated, with older users and women being more likely to provide valid details (see online supplementary figure S3). Where the Townsend score could be estimated, it showed excellent coverage of the full deprivation scale with the distribution matching well with Census data for England (figure 2).^12^

### Knowledge gaps

Information on entered numerical values was available for a 3-month period (May to July) for 138 252 users completing the user journey. Almost all users entered a plausible height (120–210 cm, 99.1%) and weight (40–200 kg, 98.2%). If users did not know their total cholesterol or BP values, national averages were applied (∼130 000 quit their data journey at this stage). From the entered data, 91.9% entered plausible total cholesterol (2–15 mmol/L) data while 8.4% entered non-plausible BP values (<50 or >250 mm Hg).

**Figure 3:**
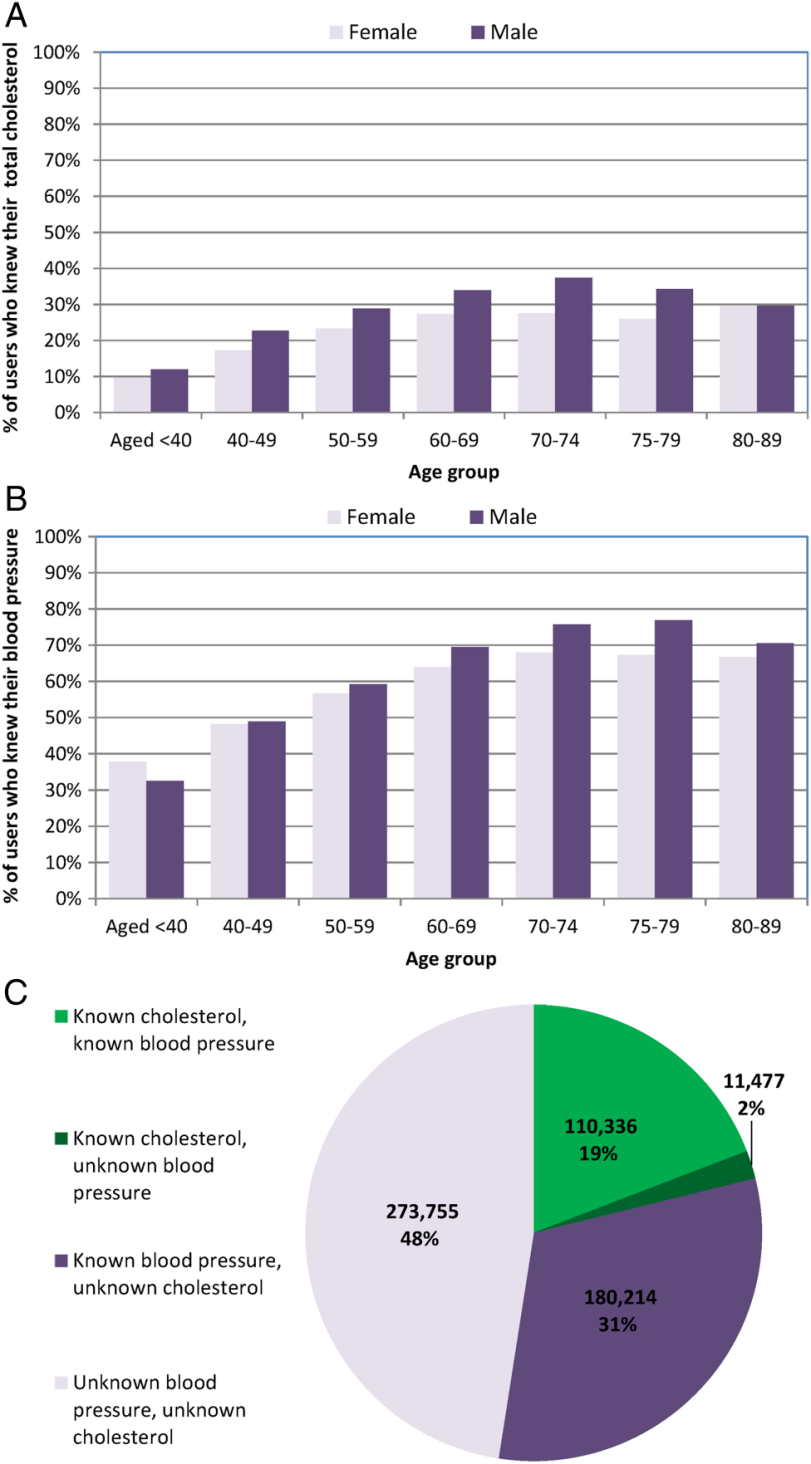
Knowledge gaps in key risk factor numbers.(A and B) Proportion by age and gender groups, who knew their total cholesterol and blood pressure numbers; (C) proportion who knew their risk factor numbers individually and in combination.

*Cholesterol*: Almost 4 out of 5 people (78.8%) did not know or input their total cholesterol values. This was slightly higher for females (81.6%) than males (77.0%) and this difference was seen for all age groups (figure 3A). Older participants (>75 years) were more likely to know their total cholesterol values compared with younger participants (<40 years; 31.1% vs 11.1%). Among those eligible for the NHS Health Check, almost three-quarters (74.3%) did not know or input their total cholesterol values.

*Blood pressure*: Half of all users (49.5%) did not enter or report knowing their SBP. This was similar in men (49.5%) and women (49.6%; figure 3B), although older users tended to know their values more compared with younger users (72.5% ages >75 years vs 34.6% ages <40 years). Among those eligible for the NHS Health Check, 42.6% did not know or enter their values. Of those who supplied their SBP, 19.3% entered 120 mm Hg exactly.

In combination, almost half of users (47.5%) did not know their BP or total cholesterol values, whereas 19.2% knew both values, and 2.0% knew their total cholesterol but not their BP levels (figure 3C).

### Risk factors and risk estimates

*Body mass index*: The mean BMI was 26.8 kg/m^2^ (SD 5.8; median 25.7 kg/m^2^, IQR 6.0); 5% were underweight (BMI<20 kg/m^2^), while 36.2% were overweight (BMI 25–29 kg/m^2^), and 21.2% obese (BMI≥30 kg/m^2^). Compared with HSE data, for each age category, height, weight and BMI were almost identical (see online supplementary figure S4).^13^

*Cholesterol*: Mean total cholesterol was 5.0 mmol/L (SD 1.3; median 5.0 mmol/L, IQR 1.5), with 43% reporting a value above this level. Both the average value and proportion with high total cholesterol (>5 mmol/L) was lower than that for the population of England, for each gender and age group, although this difference was attenuated for older users^13^ (see online supplementary figure S5).

*Blood pressure*: Mean SBP was 121.4 mm Hg (SD 20.7; median 120 mm Hg, IQR 22), with ∼9.4% reporting values >140 mm Hg. Overall, 21% either reported a SBP of >140 mm Hg or were treated for hypertension. These figures for hypertension are comparable to population estimates for England, for all age and gender groups^13^ (see online supplementary figure S6).

*Smoking*: In total, 14.4% self-reported as current smokers, while 5.3% reported having previously quit, compared with HSE 2013 estimates of 21% and 25%, respectively.^13^

*Other medical conditions*: Compared with prevalence estimates in England,^15^ self-report of the following conditions were: diabetes 4.5% (6.2%); kidney disease 1.1% (4.3%); rheumatoid arthritis 3.0% (0.7%); atrial fibrillation (AF) 2.6% (1.6%) and treated high BP 18.6% (13.7%; see online supplementary table S2).

**Figure 4:**
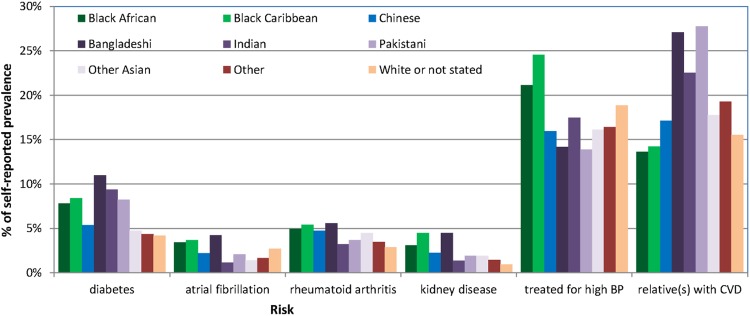
Prevalence of other medical conditions by self-reported ethnic categories. BP, blood pressure; CVD, cardiovascular disease.

*Ethnic differences*: Trends towards ethnic differences were apparent in the distribution of CVD risk factors and medical conditions. Among others, the lowest smoking rates were reported by participants identifying as Chinese (11.3%, n=681), hypertension was highest among those of Afro-Caribbean ancestry (24.5%) and diabetes was most prevalent in those of Indian ancestry (9.4%) who also had the lowest prevalence of AF (1.1%; figure 4 and see online supplementary table S2).

**Figure 5:**
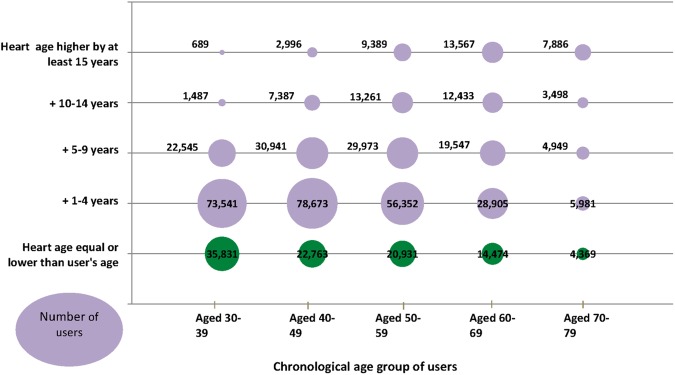
Heart age estimates with proportions who are older than their heart age, by age group of users.

*Heart age*: In total, 79.2% of all users completing the data journey through to the results page had a calculated heart age older than their chronological age, while only 8.4% were younger than their chronological age. Among younger users <40 years of age, 87% of males had a predicated heart age older than their chronological age, compared with 41% of women, despite low traditional 10-year risk estimates. Of these, 28% had a heart age greater than chronological age by at least 5 years and 14% by more than 5 years (figure 5 and see online supplementary table S3).

### Subsequent actions

After completing the data journey, 2.8% of users followed the given links on the results page, pointing to information on how to reduce specific risk factors.

## Discussion

This study, exploring the early use of the JBS3-derived heart age tool hosted on the NHS Choices website, demonstrates three key findings: (1) there is a very high level of public interest in CVD risk self-assessment when an easily understood risk metric is used; (2) many users do not know their risk factor values, emphasising the potential for NHS Health Check to fill this knowledge gap; and (3) greater use of the tool by younger users reveals a substantial and untreated risk burden, with implications for CVD prevention programmes.

Evidence from randomised studies have shown significant improvement in risk factors as well as a greater emotional impact to effect behaviour change when ‘heart age’ was communicated compared with standard metrics.^16^ ^17^ For example, Lopez-Gonzalez *et al*^16^ recently demonstrated in Spain that communication of a ‘heart age’ resulted in better smoking cessation rates, BP and weight reductions over 12 months in men and women compared with controls and those receiving standard 10-year risk estimates. This was the impetus to develop the public facing version of the JBS3 heart age calculator, to broaden access to CVD risk assessment, to empower individuals proactively to manage their risk factors and potentially to improve NHS Health Checks programme participation.^4^

There was a high uptake of the online heart age tool despite limited publicity. Within 6 months of launching, 575 000 users completed the six-page journey with the site experiencing a median of 1443 hits per day. Currently, the tool is the third most widely used application per day on the NHS Choices website, despite only being recently introduced and of much greater complexity, highlighting the potential of web-based tools for CVD prevention. Notably, there was a significant impact on uptake following media coverage,^18^ ^19^ with spikes in activity and a shift in user profiles. For example, the predominant user gender started as female but ended as male, which is unusual for this type of survey or health app. Potential explanations may include partners or family members entering data for male spouses, for example.

Valuable information is provided by the data on knowledge gaps in risk factor levels. Despite being sufficiently motivated to access and complete the tool, almost 80% of users reported not knowing their total cholesterol level and 50% a BP-level reading. A previous study of users of another online Heart Age tool derived from the Framingham risk calculator and delivered as part of the Flora/Becel marketing campaign, identified remarkably similar numbers with 77% and 47% of users not knowing their total cholesterol or BP values, respectively.^20^ Our study differs from this report as the JBS3 tool was derived from a more contemporary risk calculator (QRisk, incorporating other variables like deprivation index) and offered within the remit of the NHS as part of a wider public health campaign to encourage people to participate in the national Health Checks programme. Nonetheless the messages are consistent and figures are in line with national estimates for awareness of risk factor levels in the UK and in other countries, such as in the USA or Australia which have targeted this problem with a ‘Know Your Numbers’ campaign.^21^ These data point to an ongoing need to educate and empower the public to reduce their risk factor burden and provides support for initiatives such as the NHS Health Check programme that provide an accessible and equitable service to facilitate this drive.

Digital media often accesses a different demographic. While users of the heart age tool were broadly representative of the population of England in terms of ethnicity and deprivation categories, younger users and males were over-represented. The fact that a younger, potentially more ‘tech-savvy’ population was accessed, which is different to that attending general practitioner practices for the NHS Health Check or elsewhere is valuable and has important implications. Among those <40 years, over 69% had a heart age older than their chronological age. In particular, 28% of young males had a heart age greater than their chronological age by at least 5 years. These individuals are likely to benefit most from early CVD risk factor discussions and intervention, yet are not eligible for NHS Health Checks.

Where values were entered, height, weight and BP distributions were similar to the UK population survey data, supporting the representativeness of those accessing the tool, although overall total cholesterol, BP values and smoking prevalence appeared to be lower than expected. Prevalence of other medical conditions was broadly consistent with expected population estimates. Some ethnic differences, such as the higher prevalence of diabetes in South Asians, are well documented and support the validity of the data, while lower prevalence of AF in these same groups offers further avenues for research.

A number of refinements to the online tool are planned to understand better user characteristics, in particular where in the UK the site was accessed from, user motivation and circumstances under which it was accessed. Nevertheless, there are some limitations with the current work. First, the nature of online tools means these data are not necessarily exclusive for the UK population, although sensitivity analyses based on a valid England postcode (to indicate UK residence) did not suggest significant differences to the results presented. There is also the possibility that some users may have completed the data journey several times, for demonstration purposes or entered fictitious data. Finally high levels of ideal (eg, SBP of 120 mm Hg) or missing values could also introduce erroneous ‘heart age’ estimates, as has been shown previously.^20^ Although values are likely to be underestimated rather than overestimated with conservative default values, these data should nonetheless be interpreted with caution.

To ensure ease of use and interpretation, the NHS Choices platform did not enable individuals to determine their own potential gain by specific risk factor reduction, as offered by the full JBS3 risk tool. This may have contributed to the disappointing number (2.8%) who followed links for information on risk factor reduction, although it remains unclear how many users discussed their result with any health professionals, or sought help from other sources at a later time. Nonetheless, it is clear that there is both a need and an opportunity to leverage the public's interest in risk assessment to help initiate behaviour change, building on the heart age metric and demonstrating clear personalised opportunities for health gains from specific interventions such as smoking cessation (eg, number of life years gained by quitting, projected reduction in heart age). A number of questions remain, including the sustainability of any behavioural changes and any impact on CVD outcomes, which could potentially be addressed in the UK because of the unified healthcare system and unique patient identifiers.

In summary, there is tremendous enthusiasm from the public for self-assessment of CVD risk, when an easily understood metric is offered. Users reached by online and digital media are precisely those who would benefit most from early risk factor control and lifetime risk reduction. Many people are unaware of their own risk factors, which is a major barrier to understanding their risk, and wider knowledge is the first step towards effecting behaviour change. There is thus an important opportunity to leverage the public's interest in self-assessment of risk and combine it with traditional efforts such as the NHS Health Checks programme to reach synergistically, educate and empower the public to better understand and manage their CVD risk.

## References

[1] Townsend N, Nichols M, Scarborough P Cardiovascular disease in Europe—epidemiological update 2015. Eur Heart J 2015;36:2696–705. 10.1093/eurheartj/ehv428 26306399

[2] IDF Diabetes Atlas—7th Edition. Secondary IDF Diabetes Atlas—7th Edition 2015 http://www.diabetesatlas.org/

[3] Ng M, Fleming T, Robinson M Global, regional, and national prevalence of overweight and obesity in children and adults during 1980–2013: a systematic analysis for the Global Burden of Disease Study 2013. Lancet 2014;384:766–81. 10.1016/S0140-6736(14)60460-8 24880830 PMC4624264

[4] Putting Prevention First. Vascular checks: risk assessment and management. London: Department of Health, 2008.

[5] Fair AK, Murray PG, Thomas A Using hypothetical data to assess the effect of numerical format and context on the perception of coronary heart disease risk. Am J Health Promot 2008;22:291–6. 10.4278/061030140R2.1 18421894

[6] Cardiovascular disease: risk assessment and reduction, including lipid modification. Secondary cardiovascular disease: risk assessment and reduction, including lipid modification 2014 http://www.nice.org.uk/guidance/cg181

[7] JBS3 Board. The Joint British Societies’ consensus recommendations for the prevention of cardiovascular disease (JBS3). Heart 2014;100(Suppl 2):ii1–67. 10.1136/heartjnl-2014-305693 24667225

[8] Hippisley-Cox J, Coupland C, Robson J Derivation, validation, and evaluation of a new QRISK model to estimate lifetime risk of cardiovascular disease: cohort study using QResearch database. BMJ 2010;341:c6624 10.1136/bmj.c6624 21148212 PMC2999889

[9] Cardiovascular disease outcomes strategy: improving outcomes for people with or at risk of cardiovascular disease. London: Department of Health, 2013.

[10] NHS Choices Heart Age Tool. Secondary NHS Choices Heart Age Tool 2015 https://www.nhs.uk/tools/pages/heartage.aspx

[11] Townsend P, Phillimore P, Beattie A Health and deprivation: inequality and the north. London: Routledge, 1988.

[12] LSOA Townsend scores from unadjusted Census data England. Secondary LSOA Townsend scores from unadjusted Census data England 2001 http://www.apho.org.uk/resource/item.aspx?RID=47504

[13] Health Survey for England. Secondary Health Survey for England 2013 http://www.hscic.gov.uk/healthsurveyengland

[14] UK age and gender distribution. Secondary UK age and gender distribution 2014 http://www.ons.gov.uk/ons/taxonomy/index.html?nscl=Population+Estimates+by+Age+and+Sex

[15] HSCIC Disease Prevalence Estimates. Secondary HSCIC Disease Prevalence Estimates 2013/14. http://www.hscic.gov.uk/catalogue/PUB15751

[16] Lopez-Gonzalez AA, Aguilo A, Frontera M Effectiveness of the Heart Age tool for improving modifiable cardiovascular risk factors in a Southern European population: a randomized trial. Eur J Prev Cardiol 2015;22:389–96. 10.1177/2047487313518479 24491403

[17] Soureti A, Hurling R, Murray P Evaluation of a cardiovascular disease risk assessment tool for the promotion of healthier lifestyles. Eur J Cardiovasc Prev Rehabil 2010;17:519–23. 10.1097/HJR.0b013e328337ccd3 20195154

[18] Heart Age Tool Press Release. Secondary Heart Age Tool Press Release 2015 https://www.bhf.org.uk/health-at-work/events/newsletters/march-15/heart-age-tool

[19] When could YOU suffer a heart attack? Take this test to find out…Secondary When could YOU suffer a heart attack? Take this test to find out… 2015 http://www.dailymail.co.uk/health/article-2986128/When-suffer-heart-attack-test-out.html

[20] Neufingerl N, Cobain MR, Newson RS Web-based self-assessment health tools: who are the users and what is the impact of missing input information? J Med Internet Res 2014;16:e215 10.2196/jmir.3146 25261155 PMC4211033

[21] Cadilhac DA, Kilkenny MF, Johnson R The Know Your Numbers (KYN) program 2008 to 2010: impact on knowledge and health promotion behavior among participants. Int J Stroke 2015;10:110–16. 10.1111/ijs.12018 23490310

