# Supplementary material for: Online self-assessment of cardiovascular risk using the Joint British Societies (JBS3)-derived heart age tool: a descriptive study
Source: BMJ Open. 2016 Sep 1;6(9):e011511. doi: 10.1136/bmjopen-2016-011511 (PMC5051389; doi:10.1136/bmjopen-2016-011511)
Supplement: supplementary data [file bmjopen-2016-011511supp.pdf]

Supplementary Materials

Title: Online self-assessment of cardiovascular risk using the Joint British Societies (JBS3) derived heart age tool: A descriptive study

Running Title: Heart Age tool

Riyaz S. Patel, BHF Intermediate Fellow & Consultant Cardiologist, [1, 2]

Catherine Lagord, Public Health Analyst, [3]

Jamie Waterall, NHS Health Check National Lead, [3]

Martin Moth, Digital Tools Lead NHS Choices, [3]

Mike Knapton, Associate Medical Director BHF, [4]

John E. Deanfield, BHF Professor of Cardiology and Director NCCPO. [1, 2]

1. National Centre for Cardiovascular Prevention and Outcomes, Institute of Cardiovascular Sciences, University College London, 2nd Floor, 1 St Martin's-le-Grand, London EC1A 4NP, UK
2. Bart's Heart Centre, St Bartholomew's Hospital, West Smithfield, London, EC1A 7BE, UK
3. Public Health England, Skipton House, 80 London Road, London, SE1 6LH, UK
4. British Heart Foundation, 180 Hampstead Rd, London, NW1 7AW, UK

Contents

Supplementary Methods: 2
Supplementary Figure S1: 3
Supplementary Figure S2: 4
Supplementary Figure S3 5
Supplementary Figure S4: 6
Supplementary Figure S5 7
Supplementary Figure S6: 8
Supplementary Table S1: 9
Supplementary Table S2: 10
Supplementary Table S3: 11

Supplementary Methods:

Data items required for the heart age tool Based on QRisk

Data item	Default value
Age	30
Gender	*Not applicable*: the tool does not proceed if a gender is not selected
Ethnic group	Not stated
Townsend score	National average
Indication of already having been diagnosed with cardiovascular disease (Yes/No)	*Not applicable*: the tool does not proceed if user does not select ‘No’
Smoking status	Not a smoker
Height	*Not applicable*: the tool does not proceed if a height is not entered
Weight	*Not applicable*: the tool does not proceed if a weight is not entered
Total cholesterol level	National average for group with same age, sex and ethnicity
HDL level	National average for group with same age, sex and ethnicity
Systolic blood pressure	National average for group with same age, sex and ethnicity
Indication of ever received blood pressure treatment (Yes/No)	*Not applicable*: the tool does not proceed if no option is selected
Indication of having diabetes (Yes/No)	*Not applicable*: the tool does not proceed if no option is selected
Indication of having rheumatoid arthritis (Yes/No)	*Not applicable*: the tool does not proceed if no option is selected
Indication of having chronic kidney disease (Yes/No)	*Not applicable*: the tool does not proceed if no option is selected
Indication of atrial fibrillation (Yes/No)	*Not applicable*: the tool does not proceed if no option is selected
Indication of having close relative under 60 with CVD (Yes/No)	*Not applicable*: the tool does not proceed if no option is selected

Supplementary Figure S1: JBS3 heart age tool, website visitors by week

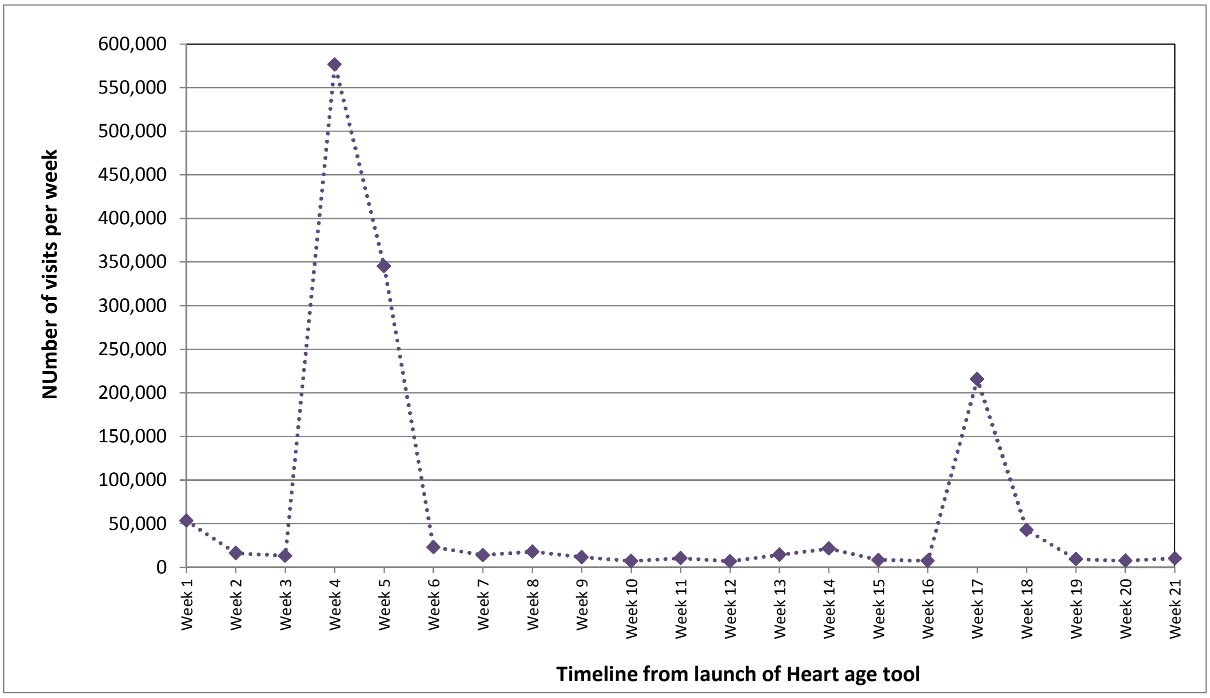

Figure demonstrating visits to the NHS choices website displaying the Heart Age tool by week. Peaks correspond to two major media coverage episodes.

Supplementary Figure S2: Gender profile of visitors completing the data journey

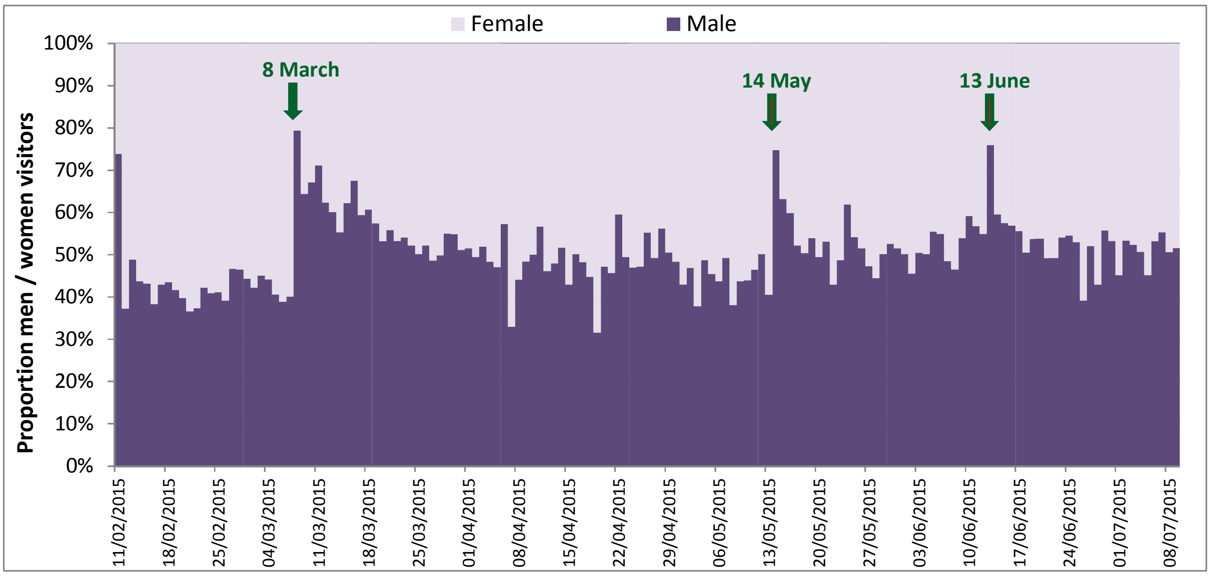

Figure illustrating changing proportion of male and female users, with shifts corresponding to media coverage (arrows).

Supplementary Figure S3: Users providing sufficient information to estimate a Townsend score

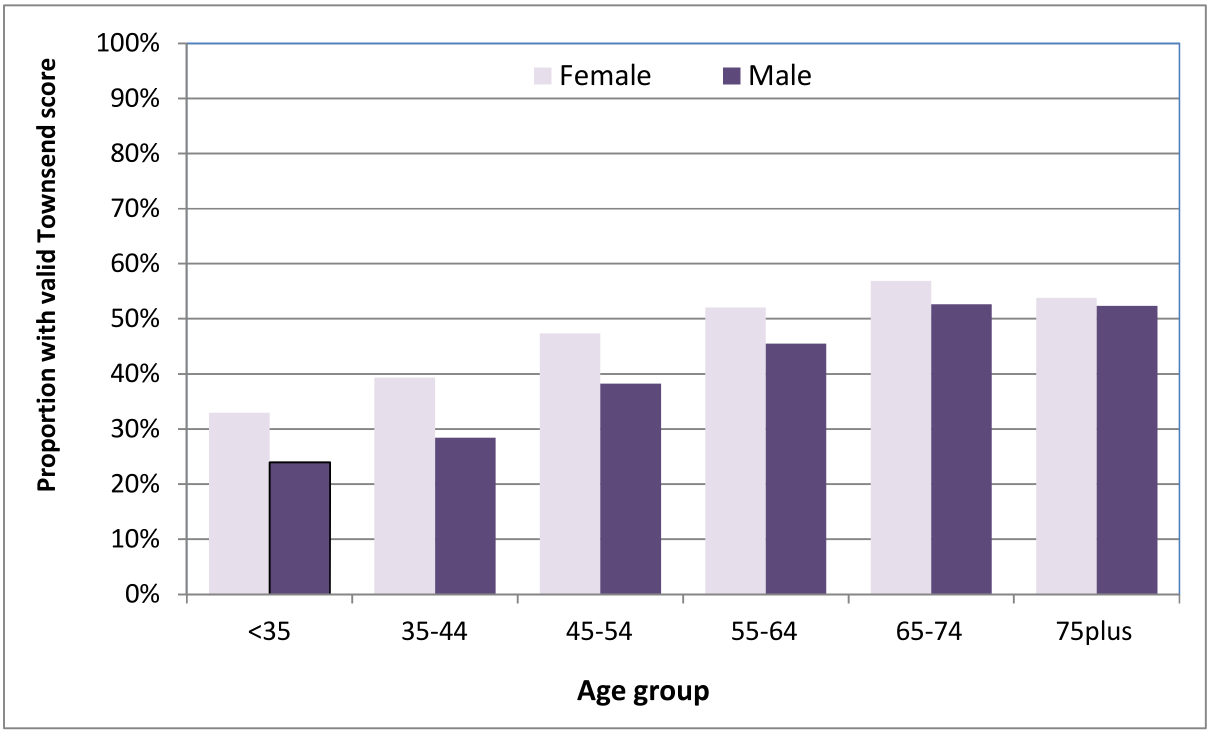

Proportion of visitors, by age and gender category who entered a valid England postcode permitting a Townsend score (deprivation index) to be calculated

Supplementary Figure S4: Height, Weight and BMI distributions compared to England population

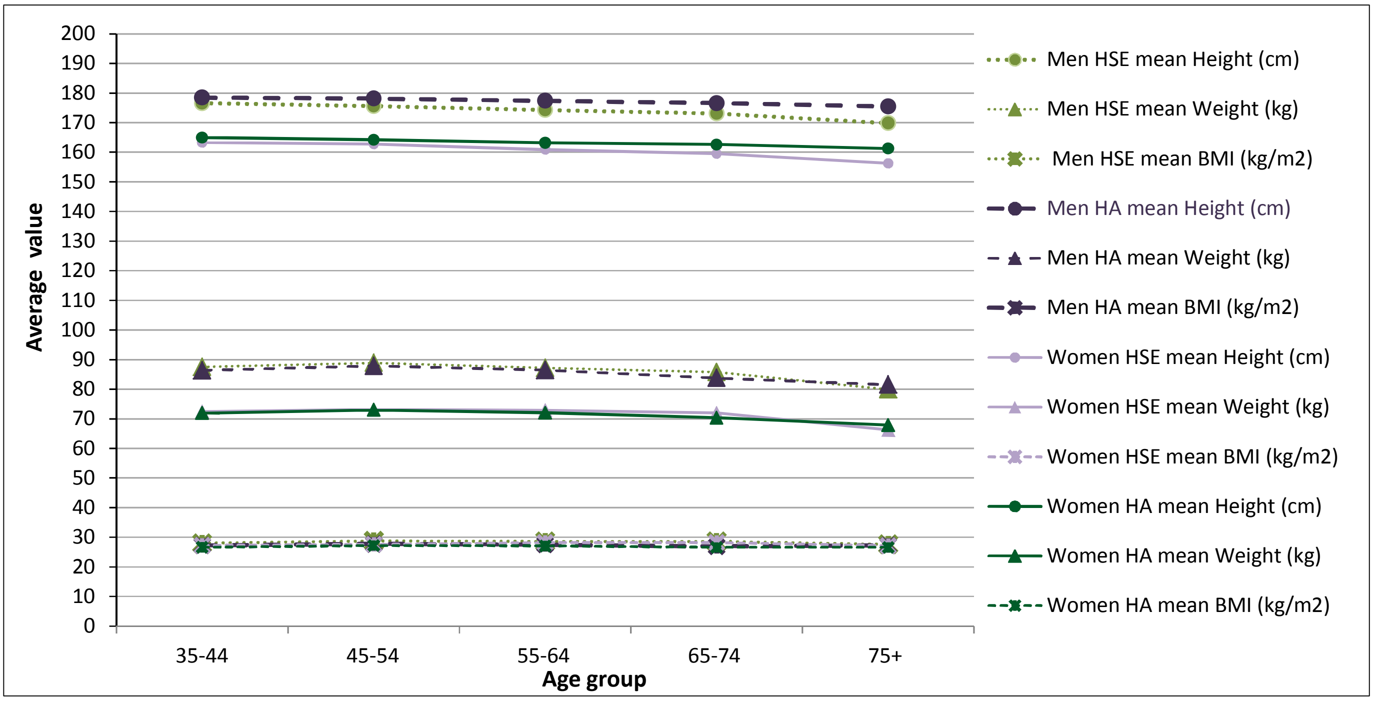

Figure illustrating distribution by age of height, weight and BMI for heart age (HA) tool users compared to data from Health Survey for England (HSE) 2013

Supplementary Figure S5: Total cholesterol values for Heart Age tool users compared to HSE survey data 2013, for men (Figure S5A) and women (Figure S5B)

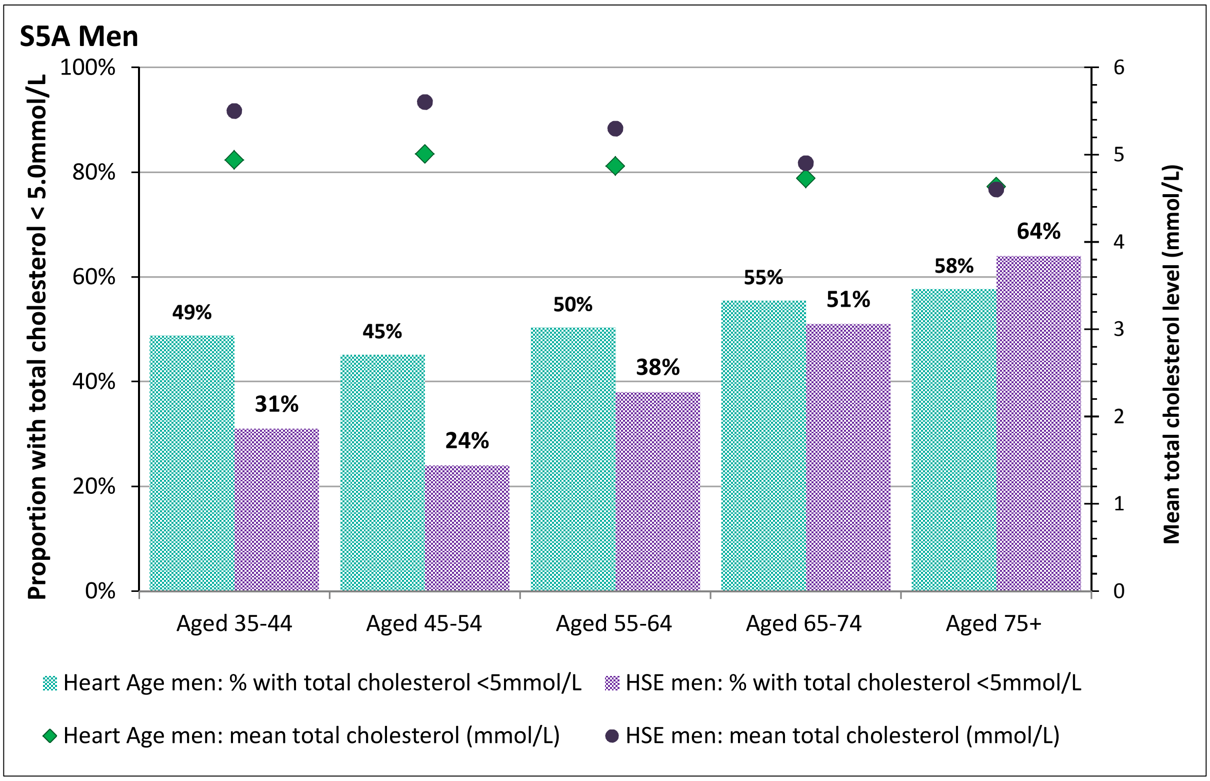

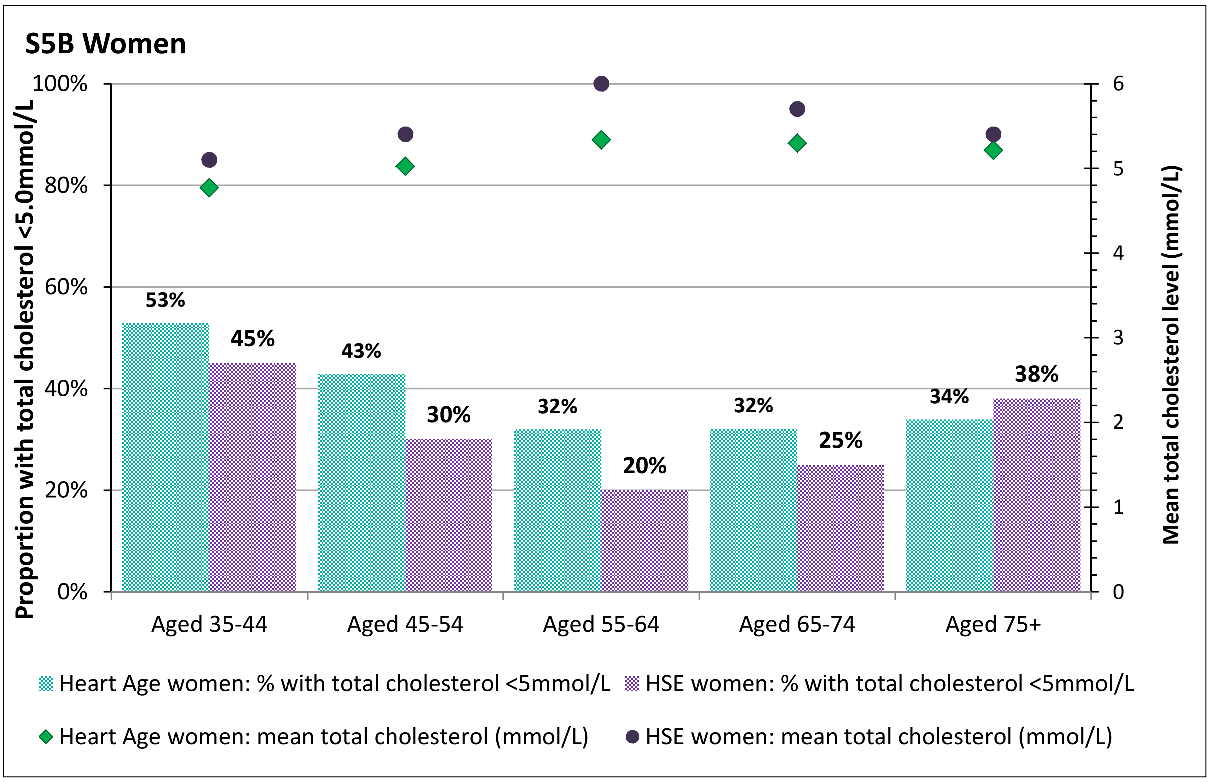

Figure illustrating distribution by age and gender for cholesterol values for heart age tool users reporting an England postcode compared to data from HSE

Supplementary Figure S6: Blood pressure values for Heart Age tool users compared to HSE survey data 2013 for men (Figure S6A) and women (Figure S6B)

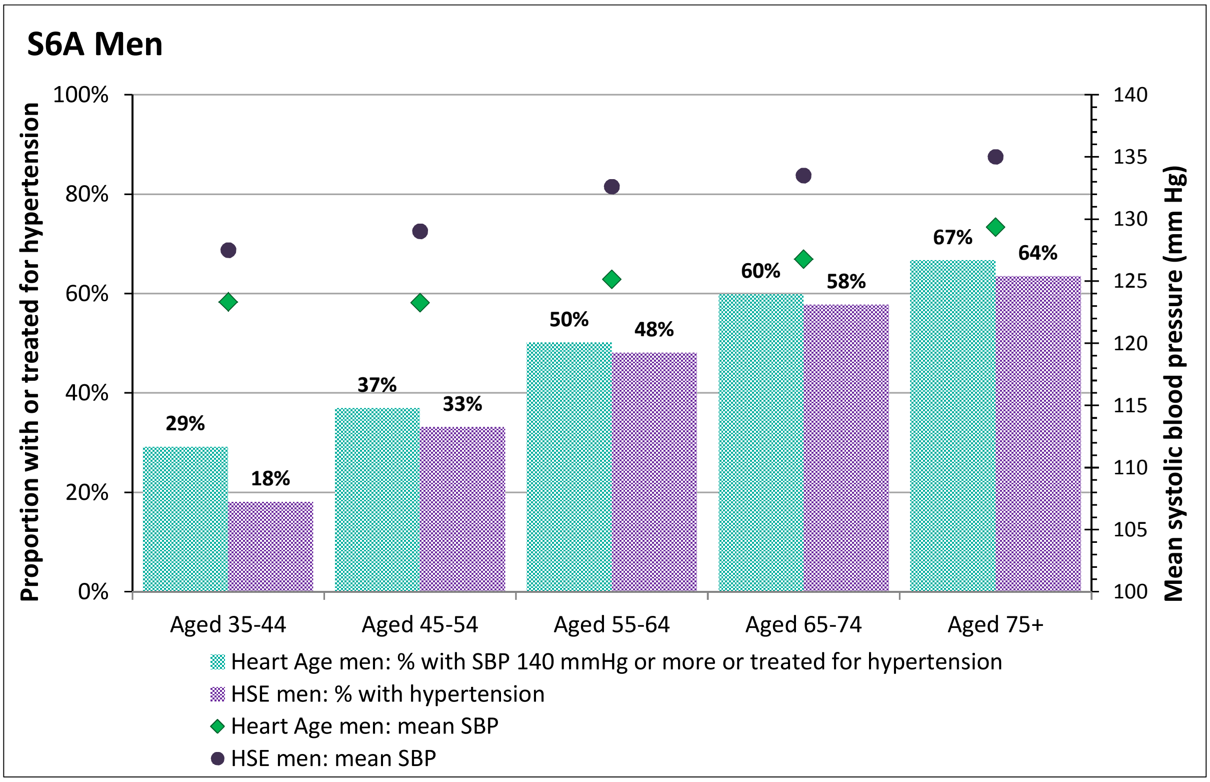

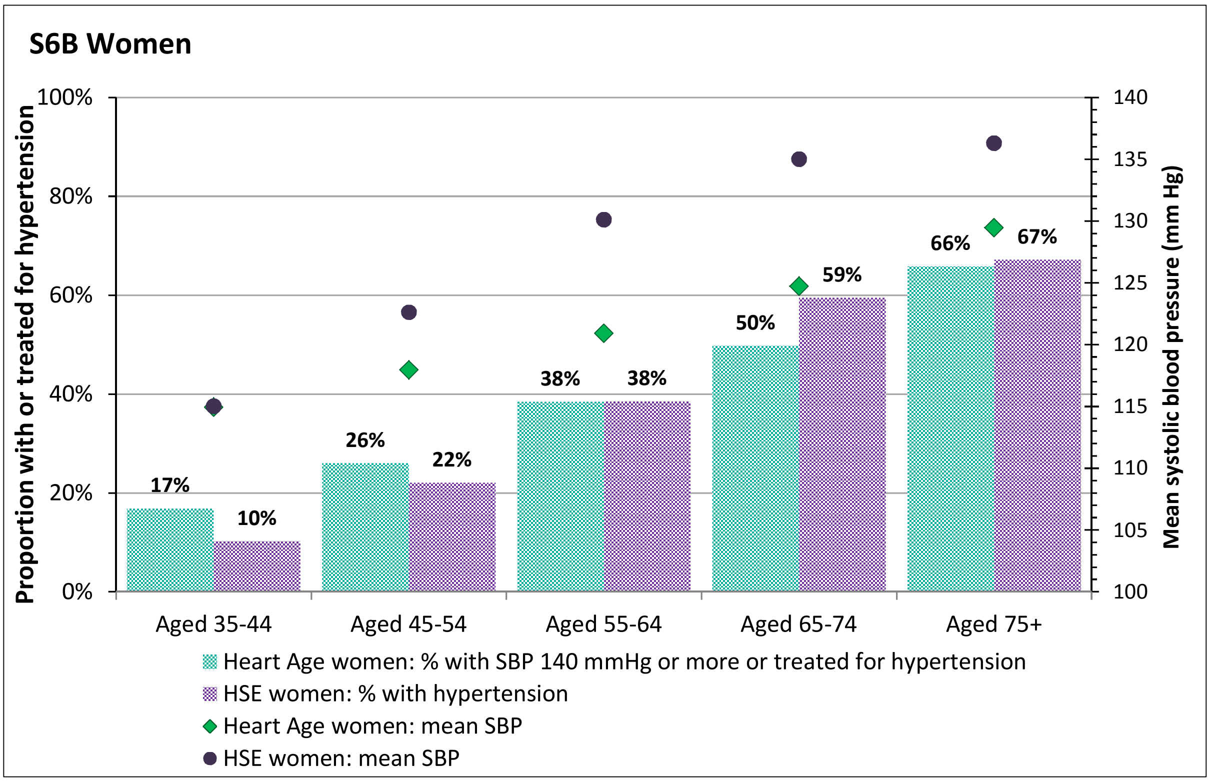

Figure illustrating distribution by age and gender for BP values for heart age tool users reporting an England postcode compared to data from HSE

Supplementary Table S1: Numbers of users completing the pathway and yielding a valid heart age

Sex and age profiles of the users completing the data journey and yielding a valid 'heart, age'

Age	Women	Men	Total
30 used as default value	19,651	29,938	49,589
30-34	29,032	46,770	75,802
35-39	21,637	36,654	58,291
40-44	28,681	43,470	72,151
45-49	29,827	40,782	70,609
50-54	30,621	39,667	70,288
55-59	25,611	34,007	59,618
60-64	20,207	28,980	49,187
65-69	14,942	24,797	39,739
70-74	6,494	12,660	19,154
75-79	2,214	5,315	7,529
80-84	711	1,839	2,550
85plus	355	920	1,275
**Total**	229,983	345,799	575,782

Supplementary Table S2: Prevalence of self-reported diseases by ethnic category

Ethnicity	Diabetes		
	no	yes	% with diabetes
Bangladeshi	1,055	130	11.0%
Black African	3,314	281	7.8%
Black Caribbean	2,116	194	8.4%
Chinese	5,702	324	5.4%
Indian	17,375	1,795	9.4%
Other	20,561	931	4.3%
Other Asian	15,723	782	4.7%
Pakistani	4,495	402	8.2%
White or not stated	479,663	20,939	4.2%
**ALL**	**550,004**	**25,778**	**4.5%**

Ethnicity	Atrial fibrillation		
	no	yes	% with atrial fibrillation
Bangladeshi	1,135	50	4.2%
Black African	3,472	123	3.4%
Black Caribbean	2,225	85	3.7%
Chinese	5,893	133	2.2%
Indian	18,951	219	1.1%
Other	21,139	353	1.6%
Other Asian	16,273	232	1.4%
Pakistani	4,796	101	2.1%
White or not stated	486,998	13,604	2.7%
**ALL**	**560,882**	**14,900**	**2.6%**

Ethnicity	Treated for high blood pressure		
	no	yes	% treated for high blood pressure
Bangladeshi	1017	168	14.2%
Black African	2835	760	21.1%
Black Caribbean	1743	567	24.5%
Chinese	5065	961	15.9%
Indian	15826	3344	17.4%
Other	17968	3524	16.4%
Other Asian	13845	2660	16.1%
Pakistani	4217	680	13.9%
White or not stated	406308	94294	18.8%
**ALL**	**468,824**	**106,958**	**18.6%**

Ethnicity	Kidney disease		
	no	yes	% with kidney disease
Bangladeshi	1,132	53	4.5%
Black African	3,484	111	3.1%
Black Caribbean	2,207	103	4.5%
Chinese	5,892	134	2.2%
Indian	18,910	260	1.4%
Other	21,187	305	1.4%
Other Asian	16,191	314	1.9%
Pakistani	4,803	94	1.9%
White or not stated	495,832	4,770	1.0%
**ALL**	**569,638**	**6,144**	**1.1%**

Ethnicity	Rheumatoid arthritis		
	no	yes	% with rheumatoid arthritis
Bangladeshi	1,119	66	5.6%
Black African	3,417	178	5.0%
Black Caribbean	2,185	125	5.4%
Chinese	5,742	284	4.7%
Indian	18,551	619	3.2%
Other	20,752	740	3.4%
Other Asian	15,767	738	4.5%
Pakistani	4,717	180	3.7%
White or not stated	486,287	14,315	2.9%
**ALL**	**558,537**	**17,245**	**3.0%**

Ethnicity	Relative under 60 with CVD		
	no	yes	% have a relative under 60 with CVD
Bangladeshi	864	321	27.1%
Black African	3105	490	13.6%
Black Caribbean	1982	328	14.2%
Chinese	4995	1031	17.1%
Indian	14852	4318	22.5%
Other	17348	4144	19.3%
Other Asian	13574	2931	17.8%
Pakistani	3538	1359	27.8%
White or not stated	422908	77694	15.5%
**ALL**	**483,166**	**92,616**	**16.1%**

Supplementary Table S3: Cross-table showing Heart Age estimates by age groups - excluding users where the default value (30) was used for chronological age

Heart age estimates by chronological age group		Heart age						Total
		Heart is younger	Heart is same age	1 to 4 years older	5 to 9 years older	10 to 14 years older	15 or more years older	
Chronological age group	30-39	6,963	28,868	73,541	22,545	1,487	689	134,093
	40-49	11,697	11,066	78,673	30,941	7,387	2,996	142,760
	50-59	13,616	7,315	56,352	29,973	13,261	9,389	129,906
	60-69	10,603	3,871	28,905	19,547	12,433	13,567	88,926
	70-79	3,235	1,134	5,981	4,949	3,498	7,886	26,683
	80-89	438	81	364	741	1,798	403	3,825
Total all chronological ages		46,552	52,335	243,816	108,696	39,864	34,930	526,193
